# Does Post-Transplant Cytomegalovirus Increase the Risk of Invasive Aspergillosis in Solid Organ Transplant Recipients? A Systematic Review and Meta-Analysis

**DOI:** 10.3390/jof7050327

**Published:** 2021-04-23

**Authors:** Nipat Chuleerarux, Achitpol Thongkam, Kasama Manothummetha, Saman Nematollahi, Veronica Dioverti-Prono, Pattama Torvorapanit, Nattapong Langsiri, Navaporn Worasilchai, Rongpong Plongla, Ariya Chindamporn, Anawin Sanguankeo, Nitipong Permpalung

**Affiliations:** 1 Department of Physiology, Faculty of Medicine, Chulalongkorn University, Bangkok 10330, Thailand; jade.negi.nipat@gmail.com; 2 Department of Microbiology, Faculty of Medicine, Chulalongkorn University, Bangkok 10330, Thailand; tachitpol@docchula.com (A.T.); kasamark.m@gmail.com (K.M.); nutleng1150@gmail.com (N.L.); drariya@gmail.com (A.C.); 3 Department of Medicine, Johns Hopkins University School of Medicine, Baltimore, MD 21205, USA; snemato1@jhmi.edu (S.N.); mdiover1@jhmi.edu (V.D.-P.); 4 Department of Medicine, Faculty of Medicine, Chulalongkorn University, King Chulalongkorn Memorial Hospital, Bangkok 10330, Thailand; pigdapaii@yahoo.com (P.T.); rongpong@hotmail.com (R.P.); 5 Faculty of Allied Health Sciences, Chulalongkorn University, Bangkok 10330, Thailand; worasilchai.navaporn@gmail.com; 6 Department of Preventive and Social Medicine, Faculty of Medicine Siriraj Hospital, Mahidol University, Bangkok 10700, Thailand; anawin_7@hotmail.com

**Keywords:** aspergillosis, CMV, cytomegalovirus, fungal infection, transplantation

## Abstract

Background: Cytomegalovirus (CMV) and invasive aspergillosis (IA) cause high morbidity and mortality in solid organ transplant (SOT) recipients. There are conflicting data with respect to the impact of CMV on IA development in SOT recipients. Methods: A literature search was conducted from existence through to 2 April 2021 using MEDLINE, Embase, and ISI Web of Science databases. This review contained observational studies including cross-sectional, prospective cohort, retrospective cohort, and case-control studies that reported SOT recipients with post-transplant CMV (exposure) and without post-transplant CMV (non-exposure) who developed or did not develop subsequent IA. A random-effects model was used to calculate the pooled effect estimate. Results: A total of 16 studies were included for systematic review and meta-analysis. There were 5437 SOT patients included in the study, with 449 SOT recipients developing post-transplant IA. Post-transplant CMV significantly increased the risk of subsequent IA with pORs of 3.31 (2.34, 4.69), I^2^ = 30%. Subgroup analyses showed that CMV increased the risk of IA development regardless of the study period (before and after 2003), types of organ transplantation (intra-thoracic and intra-abdominal transplantation), and timing after transplant (early vs. late IA development). Further analyses by CMV definitions showed CMV disease/syndrome increased the risk of IA development, but asymptomatic CMV viremia/infection did not increase the risk of IA. **Conclusions:** Post-transplant CMV, particularly CMV disease/syndrome, significantly increased the risks of IA, which highlights the importance of CMV prevention strategies in SOT recipients. Further studies are needed to understand the impact of programmatic fungal surveillance or antifungal prophylaxis to prevent this fungal-after-viral phenomenon.

## 1. Introduction

Cytomegalovirus (CMV) infection and invasive aspergillosis (IA) are important infectious complications after transplantation. CMV, like other herpesviruses, establishes lifelong latency after acute infection, which serves as a reservoir for reactivation and donor-derived infection in immunocompromised patients, including solid organ transplant (SOT) recipients and hematopoietic stem cell transplant recipients (HSCT) [1]. In SOT recipients, CMV infection or disease can occur within the first three months post-transplantation without appropriate prevention [2,3,4]. The 2019 American Society of Transplantation Infectious Diseases Community of Practice (AST IDCOP) guidelines recommended two major strategies for CMV prevention in SOT recipients: antiviral prophylaxis and preemptive therapy depending on the CMV risk profile and institution-specific protocols [5]. Despite antiviral prophylaxis with extended duration, CMV infection can occur after the completion of antiviral prophylaxis, particularly in CMV donor/recipient mismatch (D+/R−) SOT recipients. CMV infection is associated with adverse long-term outcomes, including allograft rejection, graft loss, and secondary opportunistic infections [4,6]. The mechanism behind CMV and poor clinical outcomes has been thought to be from cytopathogenicity of CMV causing direct end-organ damage and the indirect effects linked to its proinflammatory and immunosuppressive properties [7,8,9].

With regard to IA, the incidence of post-transplant IA varies among the type of organ transplantation and transplant centers [10,11,12]. The study from the Transplant Associated Infection Surveillance Network (TRANSNET) reported IA as the second most common form of invasive fungal infections (IFI) [13]. IA is associated with high rates of graft loss and mortality, with a 12-month survival of 59% [13,14]. CMV infection has been a well-described risk factor for post-transplant *Pneumocystis jirovecii* pneumonia (PJP), formerly known as *Pneumocystis carinii* pneumonia (PCP) [15,16,17]. However, there are conflicting data with respect to the impact of post-transplant CMV on subsequent IA occurrence in SOT recipients. Since both CMV and IA cause significant morbidity and mortality among SOT recipients, it is crucial to understand the interplay between these infections. Given this knowledge gap, this systematic review and meta-analysis were conducted to determine the pooled effect of post-transplant CMV on subsequent IA development in SOT populations.

## 2. Materials and Methods

### 2.1. Data Sources and Searches

We systematically searched for published studies indexed in MEDLINE (using the Ovid platform), Embase, and ISI Web of Science databases from existence through to 2 April 2021 by two authors independently (N.C. and A.T.). Search terms included cytomegalovirus, CMV, aspergillosis, organ transplantation, heart transplant, lung transplant, liver transplant, kidney transplant, pancreas transplant, small bowel transplant, small intestine transplant. Full search terms are available in the Supplementary Material (Method S). Searches from different engines were then combined, and duplicated results were deleted. A manual search for additional pertinent studies and review articles using references from retrieved articles was also completed. We contacted corresponding authors if CMV or IA definitions were not available in the study. We did not limit our search by language. The study is compliant with PRISMA (Preferred Reporting Items for Systematic reviews and Meta-Analyses) guidelines [18]. The International Prospective Register of Systematic Reviews (PROSPERO) registration number is CRD42020199227; 7 September 2020.

### 2.2. Study Selection

Two investigators (N.C. and A.T.) independently reviewed all articles. This review contained observational studies including cross-sectional, prospective cohort, retrospective cohort, and case-control studies that reported SOT with post-transplant CMV (exposure) and without post-transplant CMV (non-exposure) who developed or did not develop IA after CMV, and also presented the number of patients (%) of each group or reported measure of the association including odds ratio, hazard ratio, relative risk or risk ratio with 95% CI for developing IA. IA was defined according to the European Organization for Research and Treatment of Cancer/Mycoses Study Group (EORTC/MSG) for the diagnosis of IA [19]. Proven IA was defined by the presence of aspergillosis on microscopic analysis of sterile material, positive cultures of sterile material, or a positive fungal DNA by polymerase chain reaction combined with DNA sequencing. Probable IA was defined by the presence of a host factor (on receipt of a solid organ transplant), a clinical criterion, and mycological evidence (cytology, direct microscopy, culture, or indirect tests including detection of galactomannan antigen in plasma, serum, bronchoalveolar lavage fluid, or CSF or β-D-glucan detected in serum) [19]. IA definitions from old studies fit in with the EORTC/MSG definitions. Definitions of CMV and IA in each study are portrayed in Table 1. We excluded editorials, opinions, reviews, case reports, case series, abstract presentation, non-published studies, and duplicated or overlapped patient populations. Studies on hematologic malignancies, hematopoietic stem cell transplant, and non-transplant immunocompromised patients, including HIV, were also excluded. Study eligibility was independently determined by two investigators (N.C. and A.T.), and differences were resolved by mutual consensus or by an adjudicator (N.P.).

### 2.3. Data Extraction and Quality Assessment

We extracted data for study design, country, study year, study period, type of organ transplantation, definitions of CMV infection and IA, quantitative outcomes, study limitations, and other important comments. Our outcomes of interest were the association between post-transplant CMV and subsequent development of IA in SOT. The odds ratios (ORs), relative risks (RRs), hazard ratios (HRs), or the number of participants with the outcome of IA were collected. Non-English articles were translated with google translation during the title and abstract screening process; subsequently, they were translated by a native speaker for a full-text review. We used the Newcastle-Ottawa scale to rate the risk of bias for our review and meta-analysis since all included studies were comparative non-randomized studies [20]. This scale was divided into three parts: selection of the participants (0–4 scores), comparability between groups (0–2 scores), and the ascertainment of the outcome (0–3 scores). A total score of less than 4 was considered poor quality, 4–6 was considered moderate quality, and 7–9 was rated as high quality.

### 2.4. Data Synthesis and Analysis

We performed a meta-analysis using Comprehensive Meta-Analysis 3.3 software from Biostat, Inc. (Englewood, NJ, USA) to generate forest and funnel plots. Egger’s regression test was done by the same software. We calculated pooled effect estimates of IA outcomes with 95% confidence interval (CI) comparing SOT with and without post-transplant CMV groups using a random-effects model. We used OR as the effect estimate for this study. If OR was not available, we directly calculated unadjusted OR from quantitative data in each study. We performed sensitivity analysis by using a leave-one-out method to address potential bias [21]. Publication bias was assessed by funnel plot and Egger’s regression test [22]. The publication bias was considered significant if the *p*-value of Egger’s regression test was below 0.05 [23]. The heterogeneity of effect size estimates across these studies was quantified using the I^2^ statistic. The I^2^ statistic ranges in value from 0 to 100% (I^2^ < 25%, low heterogeneity; I^2^ = 25–60%, moderate heterogeneity; and I^2^ > 60%, substantial heterogeneity) [24].

We performed subgroups analyses to explain the heterogeneity between the studies and to examine the influence of CMV on IA in certain contexts. The following were predefined factors for subgroup analyses: CMV definitions, the timing of IA diagnosis post-transplant (early vs. late IA; early infection was defined by the average time of IA occurrence within 90 days post-transplant), type of organ transplantation (intra-abdominal vs. intra-thoracic transplantation), study period, and adjusted effect estimates (adjusted vs. unadjusted). To understand the magnitude of CMV impact on IA, we performed subgroup analyses based on CMV definitions (CMV disease/syndrome vs. asymptomatic CMV viremia/infection). We only included studies with CMV definitions consistent with the current 2019 AST IDCOP guidelines to prevent misclassification in subgroup analyses [5]. The United States Food and Drug Administration approved voriconazole for treatment of invasive fungal infections in May 2002 [25] and valganciclovir for CMV prophylaxis in high-risk populations in September 2003 [26]; hence we set a priori timepoints for the year 2003 as a surrogate for the availability of active mold azoles and CMV prevention for subgroup analyses.

**Table 1:** Study characteristics.

Study	Country	Number of Patients for Analysis	Study Design	Year of the Study	Type of Organ Transplantation	Age (Years)	CMV Definition	CMV Prophylaxis Protocols	Definition of Invasive Aspergillosis	Timing of Aspergillosis Post Transplantation (Days)
**Desbois 2016 [27]**	France	62	Case-control study	2003–2013	Kidney	IA: median 57.6 (IQR 47.7–68.2) No IA: median 56.8 (IQR 47.9–67.4)	No definition of CMV infection provided	VGCV 1.5 g was administered 4 times per day until 2006, and then VGCV 450 mg daily for 3 to 6 months.	IA was defined according to the EORTC/MSG criteria *.	Median 34 months (range 1–181 months)
**Fortún 2002 [11]**	Spain	51	Case-control study	1994–2000	Liver	IA: mean (±SD) 51 (±11) No IA: not reported	CMV disease was defined as a compatible picture associated with direct tissue culture or histologic evidence of invasive CMV disease, or when CMV viral syndrome was present; CMV infection was defined by the presence of detectable CMV by antigenemia shell vial culture of blood or by polymerase chain reaction regardless of clinical manifestation.	GCV was administered in CMV mismatch recipients for 14 days.	Proven aspergillosis: tissue histopathology showed septate, acute branching hyphae with or without a positive culture for *Aspergillus* spp. from the same site, or, in the absence of histopathology, a positive culture from tissue obtained by an invasive procedure- Probable aspergillosis: patients with a pulmonary disease with chest radiographic appearance of new nodules or cavities, and two sputum cultures or one bronchoalveolar lavage, washing, or brushing culture for *Aspergillus* spp.	Median 126 (range 22–1117)
**Fortún 2003 [28]**	Spain	280	Case-control study	1994–2001	Liver	Not reported	CMV antigenemia was defined by positive antigenemia >10 cells/200,000.	GCV was administered in CMV mismatch recipients for 14 days, followed by ACV for 3 months.	Proven aspergillosis was assigned when tissue histopathology showed septate, acute branching hyphae with or without a positive culture for Aspergillus spp. from the same site, or, in the absence of histopathology, a positive culture from tissue obtained by an invasive procedure. Probable aspergillosis applied only to patients with a pulmonary disease with chest radiographic appearance of new nodules or cavities, and two sputum cultures or one bronchoalveolar lavage, washing or brushing cultures for Aspergillus spp. In the absence of pulmonary infiltrates, the isolation of Aspergillus spp. in sputum and not confirmed in bronchoalveolar lavage was considered colonization.	Range 1–465
**Gavalda 2005 [12]**	Spain	468	Case-control study	1990–2001	Liver, kidney, kidney-pancreas, heart, and lung	IA: mean 52 (range 14–76) No IA: not reported	CMV disease was defined by consistent clinical picture associated with direct tissue culture or histological evidence of invasive CMV disease or CMV syndrome; CMV Infection was defined by detectable CMV by antigen assay and shell vial culture of blood or by PCR, regardless of clinical manifestations.	-	IA was defined according to the EORTC/MSG criteria *; only proven and probable IA was included.	Mean 234 (range 2–3025)
**He 2013 [29]**	China	28	Prospective Cohort	2005–2011	Lung	Not reported	No definition of CMV infection provided	-	IFI was defined according to the EORTC/MSG criteria *.	Median 211 (40–964)
**Heylen 2015 [30]**	Belgium	123	Case-control study	1995–2013	Kidney	IA: mean (±SD) 58 (±12) No IA: mean (±SD) 55 (±12)	No definition of CMV infection provided	GCV was given when the recipient and/or donor were CMV seropositive.	IA was defined according to the EORTC/MSG criteria *.	Median 141 (IQR 72–522 days)
**Husni 1998 [31]**	US	101	Case-control study	1990–1995	Lung	Not reported	CMV pneumonia was defined by recognition of cytomegalic inclusion bodies in tissue; CMV infection was by isolation of CMV from blood (viremia), respiratory secretions (bronchoalveolar lavage fluid), or urine in the absence of recognition of inclusion bodies in tissue. Types of CMV disease associated with IA included CMV pneumonia and CMV retinitis.	Prophylaxis for CMV infection was used for all lung transplant recipients except those with low-risk CMV (D-/R−).	Definitive IA was defined by positive culture along with positive histopathologic evidence of tissue invasion; probable pulmonary IA was defined by a characteristic clinical and radiographic picture with either histopathologic evidence of tissue invasion or culture of a respiratory tract specimen that yielded *Aspergillus*.	Mean 15 months (range 29 days–5 years)
**Kato 2014 [32]**	Japan	30	Retrospective cohort	2008–2012	Lung	IA: mean 51.4 (range 35–61) No IA: 44.2 (range 26–62)	No definition of CMV infection provided	-	IA was defined according to the EORTC/MSG criteria *.	Median 307
**López-Medrano 2016 [33]**	Spain, US, Switzerland, Belgium, Brazil, Portugal, France, Mexico, Argentina, UK	102	Case-control study	2000–2013	Kidney	IA: mean (±SD) 57.3 (±15.6) No IA: mean (±SD) 54.4 (±14.5)	CMV disease was defined by viral syndrome and probable or definitive end-organ disease.	-	IA was defined according to the EORTC/MSG criteria *.	Median 91 (IQR 65–116)
**López-Medrano 2018 [34]**	Spain, US, Switzerland, Belgium, Brazil, Portugal, France, Mexico, Argentina, UK	112	Case-control study	2000–2013	Kidney	IA: mean (±SD) 54.6 (±14.2) No IA: mean (±SD) 48.6 (±15.5)	CMV disease was defined by viral syndrome and probable or definitive end-organ disease.	-	IA was defined according to the EORTC/MSG criteria *.	Median 34.4 months (IQR 11.8–78.5 months)
**Monforte 2001 [35]**	Spain	55	Retrospective cohort	1990–1997	Lung	IA: mean 43.7 (range 15–62) No IA: mean 42.8 (range 21–67)	Diagnosis of CMV infection was based on isolation or detection of the virus from any bodily fluid or tissue specimen or antigenemia; CMV disease included CMV viral syndrome and end-organ involvement; CMV viral syndrome was defined as persistent fever, with or without leukopenia and thrombocytopenia in patients with positive blood culture or antigenemia for CMV; CMV focal disease was defined as the isolation of CMV from any tissue or body fluid plus consistent histologic findings.	GCV was administered for 15 days in all patients post-transplantation.	*Aspergillus* infection was considered when the patient had clinical symptoms, 2 or more respiratory samples were positive for *Aspergillus* spp., and at least 1 of these was obtained by bronchoscopy; invasive pulmonary aspergillosis was diagnosed when *Aspergillus* spp. was found on lung histopathology or radiologic evidence of invasion.	Mean 8.8 months (range 0.3–41 months)
**Muñoz 2004 [36]**	Spain	278	Retrospective cohort	1988–2002	Heart	IA: mean (±SD) 55 (±8.6) No IA: mean (±SD) 53 (±9.7)	CMV infection was defined by the isolation or detection of the virus from any body fluids by shell vial assay or antigenemia; CMV disease was defined by detection of signs or symptoms attributable to this microorganism and included viral syndrome and CMV focal disease.	Hyperimmunegammaglobulin and GCV were given for 15 days for CMV mismatch recipient (CMV D+/R−).	IA was defined according to the EORTC/MSG criteria *.	Median 50 ± 63
**Nagao 2016 [37]**	Japan	279	Case-control study	2007–2014	Liver	IA: mean (±SD) 51.8 (±8.8) No IA: mean (±SD) 53.5 (±10.8)	No definition of CMV infection provided	No routine CMV prophylaxis	IFI was defined according to the EORTC/MSG criteria *.	Median 79.5 (range 8–367)
**Neofytos 2018 [38]**	Switzerland	2868	Case-control study	2008–2014	Lung, heart, kidney, liver, and combined	IA: mean (±SD) 54.7 (±13.5) No IA: not reported	CMV infection and disease were defined based on the AST guidelines and the CMV definitions in transplant patients for use in a clinical trial.	-	IA was defined according to the EORTC/MSG criteria *.	Median 100 (IQR 15–275)
**Osawa 2007 [39]**	Japan	430	Case-control study	1999–2002	Liver	IA: mean (±SD) 47.5 (±4.6) No IA: mean (±SD) 44.8 (±11.7)	CMV antigenemia was defined by having at least 1 CMV pp65 antigen-positive cell/50,000 polymorphonuclear cells.	Preemptive GCV was administered in the presence of such CMV infection regardless of clinical manifestations.	IA was defined according to the EORTC/MSG criteria *.	Median 93 (range 14–333)
**Rosenhagen 2009 [40]**	Germany	170	Case-control study	2001–2004	Liver	IA: mean 54.7 (range 41–63) No IA: not reported	CMV infection was defined by positive pp65 antigenemia or at least 1 positive cell/10,000 leukocytes.	GCV was administered in CMV mismatch recipients.	IA was defined according to the EORTC/MSG criteria *.	Median 25 (range 3–282)

AST: the American Society of Transplantation; BAL: bronchoalveolar lavage; CMV: cytomegalovirus; D: donor; EORTC/MSG: the European Organization for Research and Treatment of Cancer/Mycoses Study Group; GCV: ganciclovir; GI: gastrointestinal tract; IA: invasive aspergillosis; IFI: invasive fungal infection; IQR: interquartile range; R: recipient; SD: standard deviation; US: United States of America; VGCV: valganciclovir; +: positive; −: negative. * Proven IA/IFI was defined by the presence of aspergillosis/molds on microscopic analysis of sterile material, positive cultures of sterile material, or a positive fungal DNA by polymerase chain reaction combined with DNA sequencing. Probable IA/IFI was defined by the presence of a host factor (on receipt of a solid organ), a clinical criterion, and mycological evidence (cytology, direct microscopy, culture, or indirect tests including detection of galactomannan antigen in plasma, serum, bronchoalveolar lavage fluid, or CSF or β-D-glucan detected in serum).

## 3. Results

### 3.1. Study and Patient Characteristics

**Figure 1:**
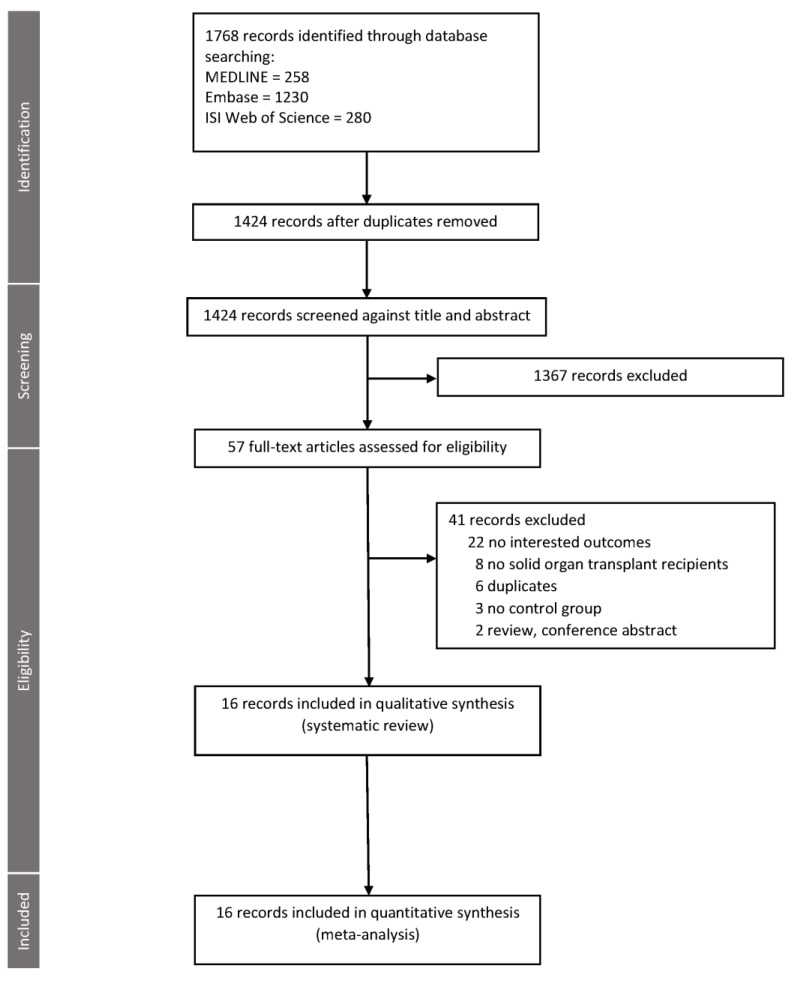
PRISMA flow chart for literature search and study selection.

Our initial search generated 1768 studies; 1367 were excluded by screening through the titles and abstracts. We performed a full-paper review with 57 articles. Forty-one articles were subsequently excluded due to no outcome of interest, no control group, or not meeting the inclusion criteria. A total of 16 studies [11,12,27,28,29,30,31,32,33,34,35,36,37,38,39,40] were included in systematic review and meta-analysis (Figure 1). The characteristics of the 16 extracted studies are described in Table 1 and Table 2. There were 5437 SOT recipients in the study, including heart, lung, liver, kidney, pancreas, kidney-pancreas, and other combined transplantation. There were 449 SOT recipients diagnosed with IA. The results of the risk of bias assessment and quality assessment are provided in the Supplementary Material (Tables S1 and S2). All studies were rated high quality.

### 3.2. Cytomegalovirus and Invasive Aspergillosis

**Figure 2:**
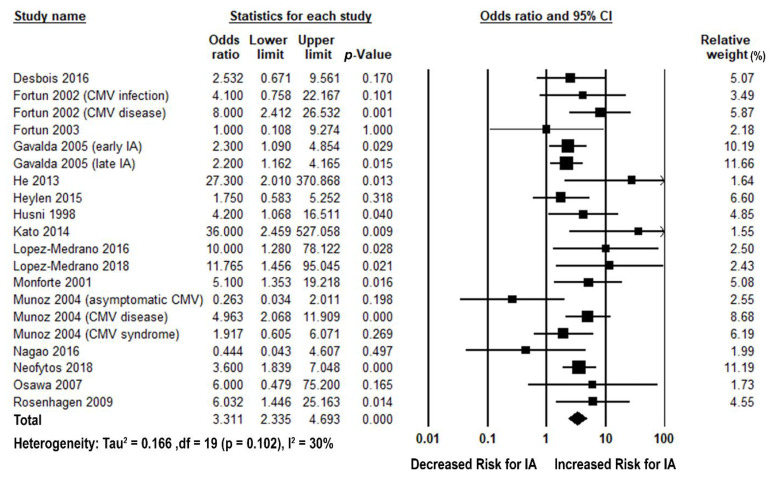
Forest plots of odds ratios for the association between CMV and post-transplant IA. CI: confidence interval; IA: invasive aspergillosis.

**Figure 3:**
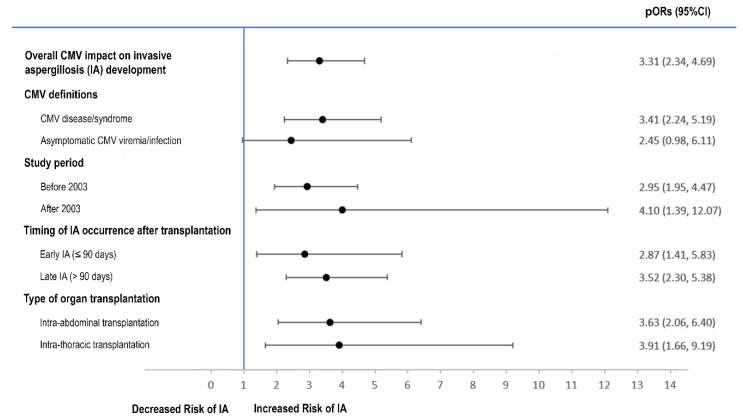
Subgroup analyses on the impact of CMV on post-transplant IA. CI: confidence interval; CMV: cytomegalovirus; IA: invasive aspergillosis; pOR: pooled odds ratio.

Sixteen studies [11,12,27,28,29,30,31,32,33,34,35,36,37,38,39,40] reported post-transplant CMV and subsequent IA outcomes among SOT recipients. CMV significantly increased the risk of post-transplant IA with a pooled odds ratio (pOR) of 3.31 (2.34, 4.69), *p* < 0.001, I^2^ = 30% (Figure 2). The sensitivity analysis by using a leave-one-out method showed significant pORs consistently (Figure S1). We observed no evidence of publication bias with the Egger test or with inspection of the funnel plots (Figure S2). Among sixteen studies reporting CMV and IA outcomes, nine used CMV definitions consistent with the AST IDCOP guidelines and were analyzed in subgroup analyses [11,12,28,31,33,34,36,38,40]. CMV disease/syndrome significantly increased the risk of subsequent IA with pOR of 3.41 (2.24, 5.19), *p*-value < 0.001, I^2^ = 21%; however, asymptomatic CMV viremia/infection did not increase the risk of IA with pOR of 2.45 (0.98, 6.11), *p*-value = 0.06, I^2^ = 49% (Figure 3 and Figure S3). Twelve studies were included for subgroup analyses by study period before and after 2003 (voriconazole/valganciclovir availability). Regardless of study period, CMV increased the risk of subsequent IA in studies conducted both before and after 2003 with pORs of 2.95 (1.95, 4.47), *p* < 0.001, I^2^ = 26% and 4.10 (1.39, 12.07), *p* < 0.001, I^2^ = 53%, respectively (Figure 3 and Figure S4).

Further subgroup analysis demonstrated that CMV increased the risk of both early and late post-transplant IA with pORs of 2.87, (1.41, 5.83), *p* = 0.004, I^2^ = 50% and 3.52 (2.30, 5.38), *p* < 0.001, I^2^ = 19%, respectively (Figure 3 and Figure S5). CMV significantly increased the risk of post-transplant IA in both intra-abdominal and intra-thoracic transplantation with pORs of 3.63 (2.06, 6.40), *p* < 0.001, I^2^ = 17% and 3.91 (1.66, 9.19), *p* = 0.002, I^2^ = 55%, respectively (Figure 3 and Figure S6). The pORs remained significant in both adjusted and unadjusted effect estimates between CMV and post-transplant IA (3.18 (1.76, 5.75), *p* < 0.001, I^2^ = 0% vs. 3.28 (2.16, 4.99), *p* < 0.001, I^2^ = 36%) (Figure S7).

This is the first systematic review and meta-analysis to demonstrate the impact of post-transplant CMV on subsequent IA occurrence in SOT. We found that post-transplant CMV significantly increased the risk of subsequent IA, regardless of the type of organ transplantation (intra-abdominal and intra-thoracic transplantation). Interestingly, CMV significantly increased the risk of both early and late IA occurrences in the SOT population. Previous studies have reported a bimodal pattern of post-transplant IA (before vs. after 90 days), suggesting that different exposures and host factors may play a role in the timing of IA occurrence [41]. Early IA, within 90 days, likely occurred in SOT recipients requiring intensive care unit level of care or dialysis after transplantation, while late IA, after 90 days, was more related to immunosuppressed states and allograft rejection [12,30]. We suspect the conflicting data on post-transplant CMV and subsequent IA in SOT is secondary to the inadequate sample size in each study, given the relatively low post-transplant IA incidence in SOT [42,43]. We further performed subgroup analyses by study period before and after 2003 as a surrogate for clinical practice changes after availability of mold active azoles and valganciclovir for CMV prophylaxis. Post-transplant CMV increased the risk of IA regardless of the study period. We believe the results confirm the association between CMV and subsequent IA in SOT. However, the results should not be interpreted as a failure of fungal prophylaxis in studies published after 2003 because it is not a common practice to start antifungal prophylaxis during or after CMV infection in SOT populations.

Potential mechanisms have been postulated to explain the inter-relationship between CMV and IA. Both CMV and IA share common risk factors such as intensified immunosuppression, rejection, and leukopenia [44,45]. CMV itself can cause leukopenia. CMV treatment-related leukopenia from intravenous ganciclovir and oral valganciclovir is well documented [46,47], both of which are first-line antiviral agents for CMV treatment and prevention [5]. Furthermore, the indirect effects of CMV infection on the host immune response have been described, which can lead to immunosuppressed states and allograft rejection, putting SOT recipients at risk for IA [7,8]. Host genetics, particularly polymorphisms in the toll-like receptor-4 (TLR-4), may play a role in increased susceptibility for both IA and CMV infections [48,49].

In this study, we further evaluated the impact of CMV on post-transplant IA based on CMV presentation. Remarkably, CMV disease/syndrome significantly increased the risk of IA, whereas asymptomatic CMV viremia/infection did not. The findings support the potential mechanisms above as CMV disease/syndrome usually presents with leukopenia, and CMV treatment, which can cause leukopenia, is almost always indicated [5]. However, these conclusions need to be interpreted with caution as only 9 out of 16 IA studies were qualified for subgroup analyses due to strict inclusion criteria by CMV definitions. This could lead to inadequate power of the impact of asymptomatic CMV viremia/infection on IA. In fact, we observed a trend towards increased risk of IA by asymptomatic CMV viremia/infection in SOT.

Even though this meta-analysis included a substantial number of studies, there are some limitations to be considered. IA and CMV shared some common risk factors; however, the current study design does not allow adjustment for all potential confounders. It is worth mentioning that the pOR from adjusted effect estimates of CMV on IA occurrence remained significant. The included studies did not provide interval duration between post-transplant CMV infection and IA development, even though all CMV events occurred prior to IA. Thus, the current study cannot evaluate the timing of IA development after CMV as well as appropriate timing/duration for both fungal surveillance and prophylaxis. Based on the results from this study, antifungal prophylaxis may be beneficial in SOT recipients with CMV, particularly CMV disease/syndrome.

In conclusion, post-transplant CMV significantly increased the risk of subsequent IA development in SOT recipients, which highlights the importance of CMV prevention strategies. Further studies on antifungal prophylaxis and other interventions for more diagnostic efforts are needed in this fungal-after-viral phenomenon.

**Table 2:** Main results of the included studies.

Study	CMV Terminology Used in the Study	Number of Cases	Incidence by Risk Exposure, Number/Total		Confounding Risk Adjustment in Multivariable Analysis	Published Measure of Association	Published Measure of Association between CMV and IA	
			IA	No IA			Univariable (95% CI)	Multivariable (95% CI)
Desbois 2016 [27]	CMV disease	16	5/16	7/46	-	-	-	-
Fortún 2002 [11]	CMV infection	13	5/13	5/38	-	OR (IA)	Overall 4.1 (0.78–22.8) Late IA 9.38 (1.21, 89.57)	-
	CMV disease	13	4/13	2/38	-	OR (IA)	Overall 8.0 (7–77) Late IA 6.38 (0.76–58.0)	-
Fortún 2003 [28]	CMV antigenemia	13	8/13	22/118	-	OR	1.0 (0.1–8.6)	-
Gavalda 2005 [12]	CMV disease	156	-	-	The effect of CMV disease for early IA development was adjusted by CMV mismatch, use of vascular amines for >24 h, additional ICU stay, post-transplantation renal failure, post-transplantation hemodialysis, >1 episode of bacterial infection, and OKT3 use	OR (IA)	Early IA 2.1 (1.1–3.8) Late IA 2.2 (1.2–4.3)	Early IA 2.3 (1.1–4.9) Late IA -
He 2013 [29]	CMV infection	8	5/7	3/21	-	OR (IA)	27.3 (2.0–369.1)	-
Heylen 2015 [30]	CMV infection	41	8/41	12/82	-	OR (IA)	1.750 (0.583–5.251)	-
Husni 1998 [31]	Cytomegalovirus disease and/or cytomegalovirus infection	14	8/14	17/57	-	OR (IA)	4.2 (1.1–17)	-
Kato 2014 [32]	CMV infection	5	3/5	1/25	-	-	-	-
López-Medrano 2016 [33]	CMV disease	51	11/51	2/51	-	OR (IA)	10.0 (1.28–78.12)	-
López-Medrano 2018 [34]	CMV disease	61	10/61	1/61	-	-	-	-
Monforte 2001 [35]	CMV disease	18	11/18	9/37	-	OR (IA)	-	5.1 (1.35–19.17)
Muñoz 2004 [36]	Asymptomatic CMV infection	24	1/24	36/254	-	-	-	-
	CMV disease	24	11/24	37/254	CMV disease was adjusted by re-operation, post-transplant hemodialysis, itraconazole prophylaxis, and another case of IA in the heart transplant program 2 months before or after the transplant date	RR (IA)	-	5.2 (2–13.9)
	CMV syndrome	24	4/24	24/254	-	-	-	-
Nagao 2016 [37]	CMV viremia	10	1/5	9/25	-	-	-	-
Neofytos 2018 [38]	CMV infection	-	-	-	-	OR (IA)	3.6 (1.8–6.9)	-
Osawa 2007 [39]	CMV infection	5	4/5	4/10	-	OR (IA)	6.0 (0.48–75.4)	-
Rosenhagen 2009 [40]	CMV infection	14	8/14	45/181	CMV infection was adjusted by dialysis, leukocytopenia, and retransplantation	OR (IA)	-	6.032 (1.446–25.163)

CI: confidence interval; CMV: cytomegalovirus; HR: hazard ratio; IA: invasive aspergillosis; ICU: intensive care unit; OR: odds ratio; RR: relative risk.

## Data Availability

The data in this article, including methods, results, and discussions, will be shared on request from the corresponding author with publication.

## Supplementary Materials

The following are available online at https://www.mdpi.com/article/10.3390/jof7050327/s1, Method S. Search strategies. Table S1. Newcastle-Ottawa quality assessment scale of included studies (cohort studies). Table S2. Newcastle-Ottawa quality assessment scale of included studies (case-control studies). Figure S1. Sensitivity analysis in invasive aspergillosis. Figure S2. Funnel plots and Egger test in invasive aspergillosis. Figure S3. Subgroup analysis in invasive aspergillosis group: CMV disease/syndrome vs. Asymptomatic CMV viremia/infection. Figure S4. Subgroup analysis in invasive aspergillosis group by study period: Before 2003 vs. After 2003. Figure S5. Subgroup analysis in invasive aspergillosis group: Early IA vs. Late IA. Figure S6. Subgroup analysis in invasive aspergillosis group: Intra-abdominal transplantation vs. Intra-thoracic transplantation. Figure S7. Subgroup analysis by adjustment of effect estimates between cytomegalovirus and invasive aspergillosis.

## Abbreviations

AST IDCOP	American Society of Transplantation Infectious Diseases Community of Practice
CI	Confidence interval
CMV	Cytomegalovirus
EORTC/MSG	European Organization for Research and Treatment of Cancer/Mycoses Study Group
HRs	Hazard ratios
HSCT	Hematopoietic stem cell transplant recipients
IA	Invasive aspergillosis
IC	Invasive candidiasis
IFI	Invasive fungal infection
ORs	Odds ratios
PCP	*Pneumocystis carinii* pneumonia
RRs	Relative risks
SOT	Solid-organ transplant
TRANSNET	Transplant Associated Infection Surveillance Network
