# Supplementary material for: Does Post-Transplant Cytomegalovirus Increase the Risk of Invasive Aspergillosis in Solid Organ Transplant Recipients? A Systematic Review and Meta-Analysis
Source: J Fungi (Basel). 2021 Apr 23;7(5):327. doi: 10.3390/jof7050327 (PMC8145336; doi:10.3390/jof7050327)
Supplement: Supplementary file 1 [file jof-07-00327-s001.zip › jof-1196687-supplementary.pdf]

Supplement Material

Method S. Search strategies

Table S1. Newcastle-Ottawa quality assessment scale of included studies (cohort studies)

Table S2. Newcastle-Ottawa quality assessment scale of included studies (case-control studies)

Figure S1. Sensitivity analysis in invasive aspergillosis

Figure S2. Funnel plots and Egger test in invasive aspergillosis

Figure S3. Subgroup analysis in invasive aspergillosis group: CMV disease/syndrome vs Asymptomatic CMV viremia/infection

Figure S4. Subgroup analysis in invasive aspergillosis group by study period: Before 2003 vs After 2003

Figure S5. Subgroup analysis in invasive aspergillosis group: Early IA vs Late IA

Figure S6. Subgroup analysis in invasive aspergillosis group: Intra-abdominal transplantation vs Intra-thoracic transplantation

Figure S7. Subgroup analysis by adjustment of effect estimates between cytomegalovirus and invasive aspergillosis

Method S. Search Strategy:

OVID Medline: retrieved 258 articles

1. Cytomegalovirus
2. exp cytomegalovirus infection
3. Cytomegalovirus.tw.
4. cmv.tw.
5. 1 or 2 or 3 or 4
6. exp Aspergillus or Aspergillus.mp.
7. Aspergillosis.mp. or exp invasive pulmonary Aspergillosis or exp Pulmonary Aspergillosis or exp Aspergillosis, Allergic Bronchopulmonary
8. Aspergill*.mp.
9. 6 or 7 or 8
10. Organ transplantation
11. exp heart transplantation
12. exp lung transplantation
13. exp kidney transplantation
14. exp liver transplantation
15. exp pancreas transplantation
16. Intestinal transplantation.mp.
17. ((Organ or heart or lung or renal or liver or pancreas or intestinal) adj transplant$).tw.
18. or/10-17
19. 5 and 9 and 18

Embase: retrieved 1,230 articles

1. 'Cytomegalovirus'/exp OR 'cytomegalovirus'
2. 'Cytomegalovirus infection'
3. cmv
4. 1 OR 2 OR 3
5. 'Aspergillosis'/exp OR 'aspergillosis'
6. 'Aspergillus'/exp OR 'aspergillus'
7. Aspergill*:ti,ab
8. 'invasive aspergillosis'/exp OR 'invasive aspergillosis'
9. 'Lung aspergillosis'/exp OR 'lung aspergillosis'
10. 5 OR 6 OR 7 OR 8 OR 9
11. 'organ transplantation'/exp OR 'organ transplantation'
12. 'heart transplantation'/exp OR 'heart transplantation'
13. 'lung transplantation'/exp OR 'lung transplantation'
14. 'kidney transplantation'/exp OR 'kidney transplantation'
15. 'liver transplantation'/exp OR 'liver transplantation'
16. 'pancreas transplantation'/exp OR 'pancreas transplantation'
17. 'intestinal transplantation'/exp OR 'intestinal transplantation'
18. (organ OR heart OR lung OR renal OR liver OR intestinal) AND transplant$
19. 11 OR 12 OR 13 OR 14 OR 15 OR 16 OR 17 OR 18
20. 4 AND 10 AND 19

ISI Web of Science: retrieved 280 articles

1. ALL=Cytomegalovirus
2. ALL=(cytomegalovirus infection*)
3. ALL=cmv
4. 1 OR 2 OR 3
5. ALL=Aspergillus
6. ALL=Aspergillosis
7. ALL=Aspergill*
8. ALL=(Pulmonary aspergill*)
9. 5 OR 6 OR 7 OR 8
10. ALL=(Organ transplantation*)
11. ALL=(Heart transplantation*)
12. ALL=(Lung transplantation*)
13. ALL=((Kidney OR Renal) AND (transplantation*))
14. ALL=(Liver transplantation*)
15. ALL=(Pancreas transplantation*)
16. ALL=((Intestine OR Intestinal OR Small bowel) AND (transplantation*))
17. 10 OR 11 OR 12 OR 13 OR 14 OR 15 OR 16
18. 4 and 9 and 17

Table S1. Newcastle-Ottawa quality assessment scale of included cohort studies

Study/Year	Selection				Comparability	Outcome			Total score
	Representativeness	Selection of the non-exposed cohort	Ascertainment	Endpoint does not present at start	Comparability (Confounding)	Assessment of outcome	Follow-up duration	Adequacy follow-up	
**He, 2013**	*	*	*	*		*	*	*	7
**Kato, 2014**	*	*		*	**	*	*	*	8
**Monforte, 2001**	*	*		*	**	*	*	*	8
**Munoz, 2004**	*	*		*	**	*	*	*	8

Table S2. Newcastle-Ottawa quality assessment scale of included case-control studies

Study/Year	Selection				Comparability	Exposure			Total score
	Case Definition	Representativeness	Selection of Controls	Definition of Controls	Comparability (Confounding)	Ascertainment of exposure	Ascertainment method	Non-respons e rate	
**Desbois, 2016**	*	*	*	*	**	*	*	*	9
**Fortun, 2002**	*	*	*	*	**	*	*	*	9
**Fortun, 2003**	*	*	*	*	**			*	7
**Gavalda, 2005**	*	*	*	*	*	*	*	*	8
**Heylen, 2015**	*	*	*	*	**	*	*	*	9
**Husni, 1998**	*	*	*	*		*	*	*	7
**Lopez-Medrano, 2016**	*	*	*	*	**	*	*	*	9
**Lopez-Medrano, 2018**	*	*	*	*	**	*	*	*	9
**Nagao, 2016**		*	*	*	**	*	*	*	8
**Neofytos, 2018**	*	*	*	*	**	*	*	*	9
**Osawa, 2007**	*	*	*	*	**			*	7
**Rosenhagen, 2009**	*	*	*	*		*	*	*	7

Figure S1. Sensitivity analysis in invasive aspergillosis

Study name	Point	Lower limit	Upper limit	p-Value
Desbois 2016	3.373	2.333	4.877	0.000
Fortun 2002 (CMV infection)	3.296	2.289	4.747	0.000
Fortun 2002 (CMV disease)	3.124	2.199	4.439	0.000
Fortun 2003	3.401	2.388	4.844	0.000
Gavalda 2005 (early IA)	3.462	2.369	5.061	0.000
Gavalda 2005 (late IA)	3.500	2.395	5.115	0.000
He 2013	3.186	2.270	4.471	0.000
Heylen 2015	3.468	2.409	4.991	0.000
Husni 1998	3.283	2.273	4.742	0.000
Kato 2014	3.174	2.273	4.432	0.000
Lopez-Medrano 2016	3.217	2.261	4.579	0.000
Lopez-Medrano 2018	3.205	2.258	4.548	0.000
Monforte 2001	3.243	2.250	4.675	0.000
Munoz 2004 (asymptomatic CMV)	3.429	2.516	4.674	0.000
Munoz 2004 (CMV disease)	3.195	2.204	4.633	0.000
Munoz 2004 (CMV syndrome)	3.441	2.385	4.965	0.000
Nagao 2016	3.421	2.438	4.800	0.000
Neofytos 2018	3.304	2.239	4.877	0.000
Osawa 2007	3.284	2.293	4.702	0.000
Rosenhagen 2009	3.222	2.244	4.626	0.000
**Total**	3.311	2.335	4.693	0.000

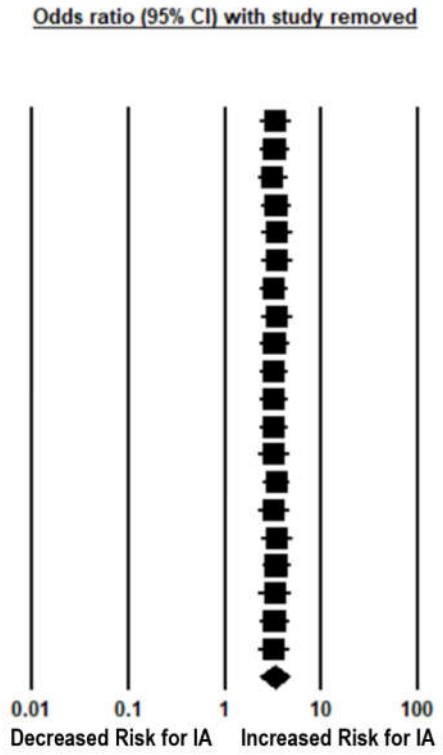

CI: confidence interval; CMV: Cytomegalovirus; IA: invasive aspergillosis

Figure S2. Funnel plots and Egger test in invasive aspergillosis

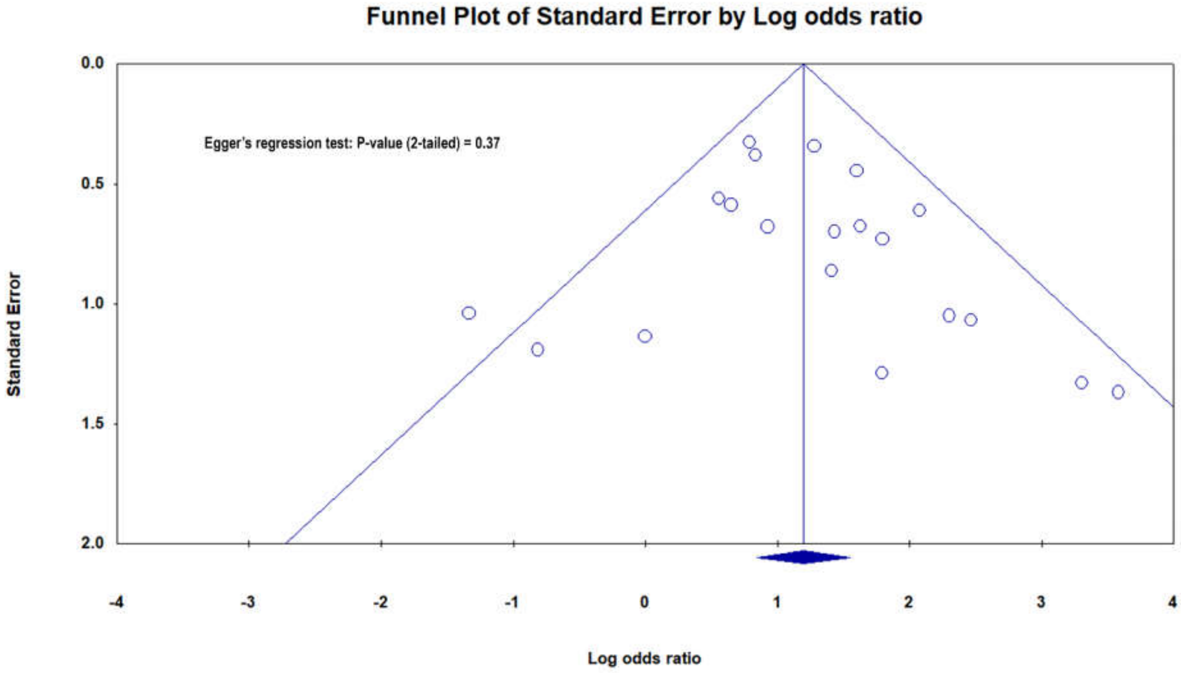

Figure S3. Subgroup analysis in invasive aspergillosis group: CMV disease/syndrome vs Asymptomatic CMV viremia/infection

CMV disease/syndrome

Study name	Odds ratio	Lower limit	Upper limit	p-Value	Relative weight (%)
Fortun 2002 (CMV disease)	8.000	2.412	26.532	0.001	10.23
Gavalda 2005 (early IA)	2.300	1.090	4.854	0.029	20.86
Gavalda 2005 (late IA)	2.200	1.162	4.165	0.015	25.37
Husni 1998	4.200	1.068	16.511	0.040	8.17
Lopez-Medrano 2016	10.000	1.280	78.122	0.028	3.91
Lopez-Medrano 2018	11.765	1.456	95.045	0.021	3.80
Munoz 2004 (CMV disease)	4.963	2.068	11.909	0.000	16.74
Munoz 2004 (CMV syndrome)	1.917	0.605	6.071	0.269	10.92
**Total**	3.410	2.240	5.192	0.000	

Statistics for each study

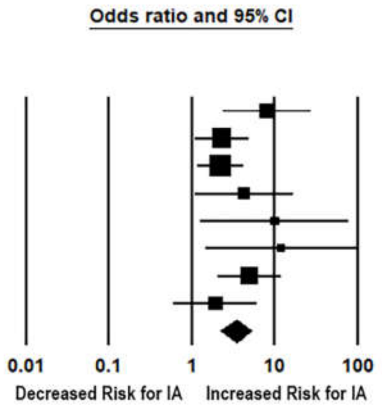

Heterogeneity: $Tau^2 = 0.075$,df = 7 ($p = 0.262$), $I^2 = 21\%$

Asymptomatic CMV viremia/infection

Study name	Odds ratio	Lower limit	Upper limit	p-Value	Relative weight (%)
Fortun 2002 (CMV infection)	4.100	0.758	22.167	0.101	17.55
Fortun 2003	1.000	0.108	9.274	1.000	12.16
Munoz 2004 (asymptomatic CMV)	0.263	0.034	2.011	0.198	13.83
Neofytos 2018	3.600	1.839	7.048	0.000	35.32
Rosenhagen 2009	6.032	1.446	25.163	0.014	21.14
**Total**	2.448	0.981	6.111	0.055	

Statistics for each study

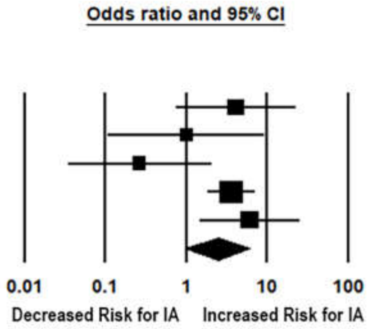

Heterogeneity: $Tau^2 = 0.499$,df = 4 ($p = 0.100$), $I^2 = 49\%$

CI: confidence interval; CMV: Cytomegalovirus; IA: invasive aspergillosis

Figure S4. Subgroup analysis in invasive aspergillosis group by study period: Before 2003 vs After 2003

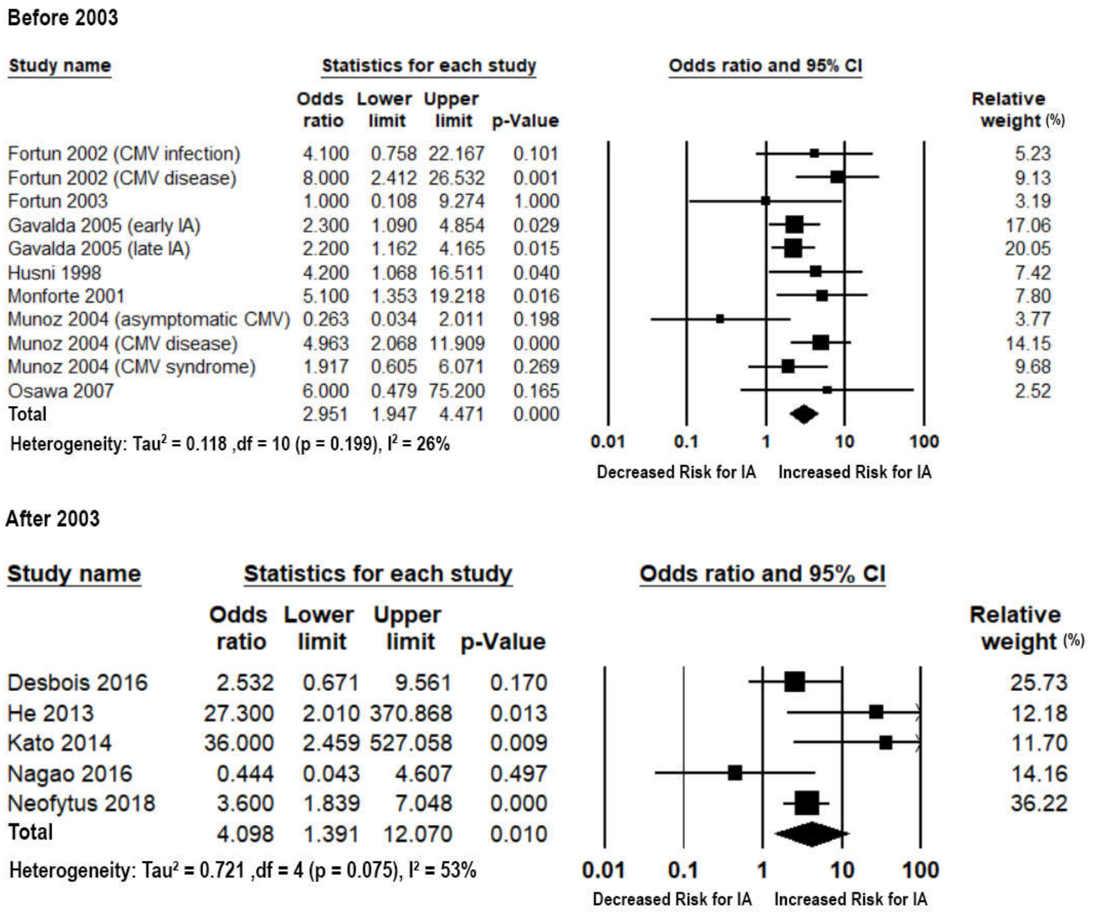

CI: confidence interval; IA: invasive aspergillosis

Figure S5. Subgroup analysis in invasive aspergillosis group: Early IA vs Late IA

Early IA

Study name	Odds ratio	Lower limit	Upper limit	p-Value	Relative weight (%)
Gavalda 2005 (early IA)	2.300	1.090	4.854	0.029	25.68
Lopez-Medrano 2016	10.000	1.280	78.122	0.028	8.95
Munoz 2004 (asymptomatic CMV)	0.263	0.034	2.011	0.198	9.10
Munoz 2004 (CMV disease)	4.963	2.068	11.909	0.000	23.21
Munoz 2004 (CMV syndrome)	1.917	0.605	6.071	0.269	18.43
Rosenhagen 2009	6.032	1.446	25.163	0.014	14.63
**Total**	2.867	1.410	5.830	0.004	

Heterogeneity: Tau^2 = 0.365 ,df = 5 (p = 0.073), I^2 = 50%

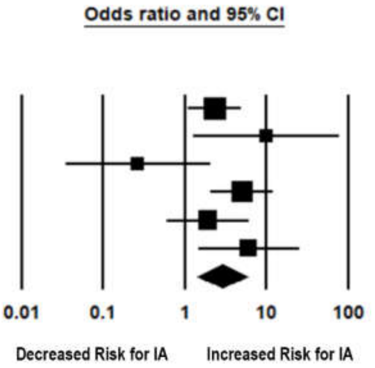

Late IA

Study name	Odds ratio	Lower limit	Upper limit	p-Value	Relative weight (%)
Desbois 2016	2.532	0.671	9.561	0.170	8.29
Fortun 2002 (CMV infection, late IA)	9.380	1.090	80.703	0.041	3.58
Fortun 2002 (CMV disease, late IA)	6.380	0.730	55.735	0.094	3.53
Gavalda 2005 (late IA)	2.200	1.162	4.165	0.015	22.01
He 2013	27.300	2.010	370.868	0.013	2.50
Heylen 2015	1.750	0.583	5.252	0.318	11.14
Husni 1998	4.200	1.068	16.511	0.040	7.89
Kato 2014	36.000	2.459	527.058	0.009	2.37
Lopez-Medrano 2018	11.765	1.456	95.045	0.021	3.78
Monforte 2001	5.100	1.353	19.218	0.016	8.31
Nagao 2016	0.444	0.043	4.607	0.497	3.07
Neofytos 2018	3.600	1.839	7.048	0.000	20.88
Osawa 2007	6.000	0.479	75.200	0.165	2.65
**Total**	3.518	2.300	5.381	0.000	

Heterogeneity: Tau^2 = 0.107 ,df = 12 (p = 0.249), I^2 = 19%

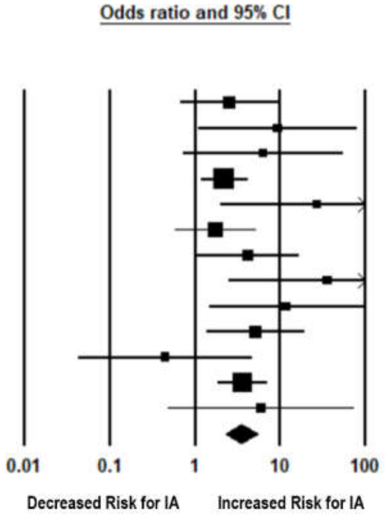

CI: confidence interval; IA: invasive aspergillosis

Figure S6. Subgroup analysis in invasive aspergillosis group: Intra-abdominal transplantation vs Intra-thoracic transplantation

Intra-abdominal transplantation

Study name	Odds ratio	Lower limit	Upper limit	p-Value	Relative weight (%)
Desbois 2016	2.532	0.671	9.561	0.170	13.99
Fortun 2002 (CMV infection)	4.100	0.758	22.167	0.101	9.52
Fortun 2002 (CMV disease)	8.000	2.412	26.532	0.001	16.31
Fortun 2003	1.000	0.108	9.274	1.000	5.86
Heylen 2015	1.750	0.583	5.252	0.318	18.46
Lopez-Medrano 2016	10.000	1.280	78.122	0.028	6.76
Lopez-Medrano 2018	11.765	1.456	95.045	0.021	6.57
Nagao 2016	0.444	0.043	4.607	0.497	5.36
Osawa 2007	6.000	0.479	75.200	0.165	4.65
Rosenhagen 2009	6.032	1.446	25.163	0.014	12.50
**Total**	3.630	2.057	6.403	0.000	

Heterogeneity: $Tau^2 = 0.140$, df = 9 ($p = 0.287$), $I^2 = 17\%$

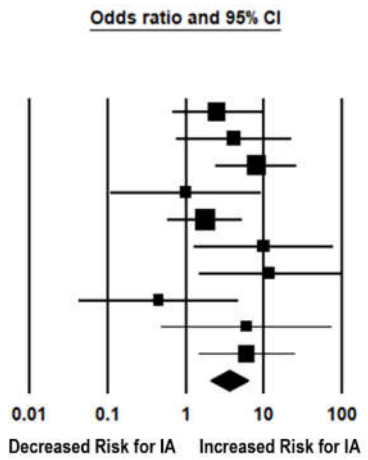

Intra-thoracic transplantation

Study name	Odds ratio	Lower limit	Upper limit	p-Value	Relative weight (%)
He 2013	27.300	2.010	370.868	0.013	7.79
Husni 1998	4.200	1.068	16.511	0.040	16.42
Kato 2014	36.000	2.459	527.058	0.009	7.48
Monforte 2001	5.100	1.353	19.218	0.016	16.85
Munoz 2004 (asymptomatic CMV)	0.263	0.034	2.011	0.198	10.90
Munoz 2004 (CMV disease)	4.963	2.068	11.909	0.000	21.85
Munoz 2004 (CMV syndrome)	1.917	0.605	6.071	0.269	18.71
**Total**	3.905	1.660	9.185	0.002	

Heterogeneity: $Tau^2 = 0.672$, df = 6 ($p = 0.037$), $I^2 = 55\%$

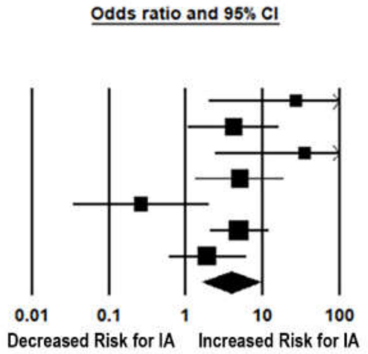

CI: confidence interval; IA: invasive aspergillosis

Figure S7. Subgroup analysis by adjustment of effect estimates between cytomegalovirus and invasive aspergillosis

Adjusted effect estimates between CMV and IA

Study name	Odds ratio	Lower limit	Upper limit	p-Value	Relative weight (%)
Gavalda 2005 (early IA)	2.300	1.090	4.854	0.029	62.87
Monforte 2001	5.100	1.353	19.218	0.016	19.93
Rosenhagen 2009	6.032	1.446	25.163	0.014	17.20
**Total**	3.182	1.760	5.753	0.000	

Heterogeneity: Tau² = 0.000 ,df = 2 (p = 0.371), I² = 0%

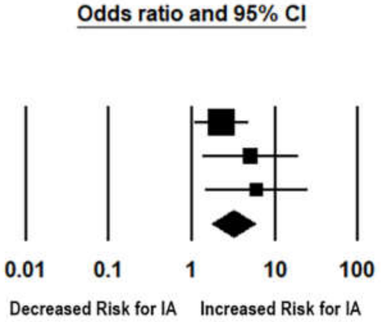

Unadjusted effect estimates between CMV and IA

Study name	Odds ratio	Lower limit	Upper limit	p-Value	Relative weight (%)
Desbois 2016	2.532	0.671	9.561	0.170	6.53
Fortun 2002 (CMV infection)	4.100	0.758	22.167	0.101	4.65
Fortun 2002 (CMV disease)	8.000	2.412	26.532	0.001	7.43
Fortun 2003	1.000	0.108	9.274	1.000	2.98
Gavalda 2005 (late IA)	2.200	1.162	4.165	0.015	13.18
He 2013	27.300	2.010	370.868	0.013	2.27
Heylen 2015	1.750	0.583	5.252	0.318	8.23
Husni 1998	4.200	1.068	16.511	0.040	6.27
Kato 2014	36.000	2.459	527.058	0.009	2.16
Lopez-Medrano 2016	10.000	1.280	78.122	0.028	3.41
Lopez-Medrano 2018	11.765	1.456	95.045	0.021	3.32
Munoz 2004 (asymptomatic CMV)	0.263	0.034	2.011	0.198	3.47
Munoz 2004 (CMV disease)	4.963	2.068	11.909	0.000	10.38
Munoz 2004 (CMV syndrome)	1.917	0.605	6.071	0.269	7.79
Nagao 2016	0.444	0.043	4.607	0.497	2.75
Neofytos 2018	3.600	1.839	7.048	0.000	12.76
Osawa 2007	6.000	0.479	75.200	0.165	2.40
**Total**	3.283	2.159	4.992	0.000	

Heterogeneity: Tau² = 0.241 ,df = 16 (p = 0.068), I² = 36%

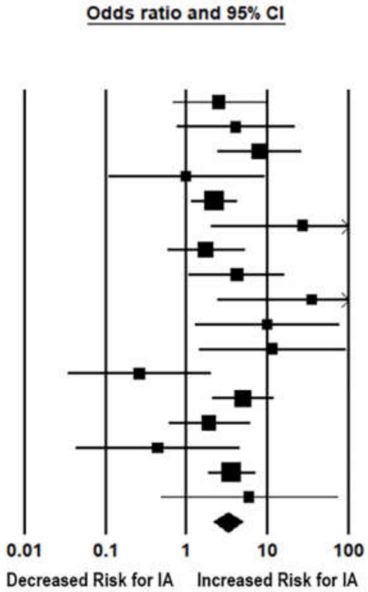

CI: confidence interval; CMV: Cytomegalovirus; IA: invasive aspergillosis
